# Vaccine effectiveness against COVID-19 hospitalisation in adults (≥ 20 years) during Alpha- and Delta-dominant circulation: I-MOVE-COVID-19 and VEBIS SARI VE networks, Europe, 2021

**DOI:** 10.2807/1560-7917.ES.2023.28.47.2300186

**Published:** 2023-11-23

**Authors:** Angela MC Rose, Nathalie Nicolay, Virginia Sandonis Martín, Clara Mazagatos, Goranka Petrović, F Annabel Niessen, Ausenda Machado, Odile Launay, Sarah Denayer, Lucie Seyler, Joaquin Baruch, Cristina Burgui, Isabela I Loghin, Lisa Domegan, Roberta Vaikutytė, Petr Husa, George Panagiotakopoulos, Nassera Aouali, Ralf Dürrwald, Jennifer Howard, Francisco Pozo, Bartolomé Sastre-Palou, Diana Nonković, Mirjam J Knol, Irina Kislaya, Liem binh Luong Nguyen, Nathalie Bossuyt, Thomas Demuyser, Aušra Džiugytė, Iván Martínez-Baz, Corneliu Popescu, Róisín Duffy, Monika Kuliešė, Lenka Součková, Stella Michelaki, Marc Simon, Janine Reiche, María Teresa Otero-Barrós, Zvjezdana Lovrić Makarić, Patricia CJL Bruijning-Verhagen, Verónica Gomez, Zineb Lesieur, Cyril Barbezange, Els Van Nedervelde, Maria-Louise Borg, Jesús Castilla, Mihaela Lazar, Joan O’Donnell, Indrė Jonikaitė, Regina Demlová, Marina Amerali, Gil Wirtz, Kristin Tolksdorf, Marta Valenciano, Sabrina Bacci, Esther Kissling, I-MOVE-COVID-19 hospital study team, VEBIS hospital study team, Members of the I-MOVE-COVID-19 and VEBIS hospital study teams (in addition to the named authors), Svjetlana Karabuva, Petra Tomaš Petrić, Marija Marković, Sandra Ljubičić, Bojana Mahmutović, Irena Tabain, Petra Smoljo, Iva Pem Novosel, Tanya Melillo, John Paul Cauchi, Benédicte Lissoir, Xavier Holemans, Marc Hainaut, Nicolas Dauby, Benedicte Delaere, Marc Bourgeois, Evelyn Petit, Marijke Reynders, Door Jouck, Koen Magerman, Marieke Bleyen, Melissa Vermeulen, Sébastien Fierens, François Dufrasne, Siel Daelemans, Ala’a Al Kerwi, Francoise Berthet, Guy Fagherazzi, Myriam Alexandre, Charlene Bennett, Jim Christle, Jeff Connell, Peter Doran, Laura Feeney, Binita Maharjan, Sinead McDermott, Rosa McNamara, Nadra Nurdin, Salif Mamadou Cissé, Anne-Sophie L'Honneur, Xavier Duval, Yolande Costa, Fidouh Nadhira, Florence Galtier, Laura Crantelle, Vincent Foulongne, Phillipe Vanhems, Sélilah Amour, Bruno Lina, Fabrice Lainé, Laetitia Gallais, Gisèle Lagathu, Anna Maisa, Yacine Saidi, Christine Durier, Rebecca Bauer, Ana Paula Rodrigues, Adriana Silva, Raquel Guiomar, Margarida Tavares, Débora Pereira, Maria José Manata, Heidi Gruner, André Almeida, Paula Pinto, Cristina Bárbara, Itziar Casado, Ana Miqueleiz, Ana Navascués, Camino Trobajo-Sanmartín, Miguel Fernández-Huerta, María Eugenia Portillo, Carmen Ezpeleta, Nerea Egüés, Manuel García Cenoz, Eva Ardanaz, Marcela Guevara, Conchi Moreno-Iribas, Hana Orlíková, Carmen Mihaela Dorobat, Carmen Manciuc, Simin Aysel Florescu, Alexandru Marin, Sorin Dinu, Catalina Pascu, Alina Ivanciuc, Iulia Bistriceanu, Mihaela Oprea, Maria Elena Mihai, Silke Buda, Ute Preuss, Marianne Wedde, Auksė Mickienė, Giedrė Gefenaitė, Alain Moren, Anthony Nardone

**Affiliations:** 1 Epiconcept, Paris, France; 2 European Centre for Disease Prevention and Control, Stockholm, Sweden; 3 National Centre for Microbiology, Institute of Health Carlos III, Madrid, Spain; 4 Consortium for Biomedical Research in Epidemiology and Public Health (CIBERESP), Madrid, Spain; 5 National Centre for Epidemiology, Institute of Health Carlos III, Madrid, Spain; 6 Croatian Institute of Public Health, Zagreb, Croatia; 7 Centre for Infectious Disease Control, National Institute for Public Health and the Environment, Bilthoven, the Netherlands; 8 National Institute of Health Dr Ricardo Jorge, Lisbon, Portugal; 9 Faculty of Medicine, University of Paris City, Paris, France; 10 AP–HP, Hôpital Cochin, Paris, France; 11 Inserm, CIC Cochin-Pasteur, Paris, France; 12 Sciensano, Brussels, Belgium; 13 Universitair Ziekenhuis Brussel, Brussels, Belgium; 14 IDCU within Health promotion and disease prevention Directorate, G’mangia, Malta; 15 Instituto de Salud Pública de Navarra-IdiSNA, Pamplona, Spain; 16 Grigore T. Popa University of Medicine and Pharmacy, Iasi, Romania; 17 St. Parascheva Clinical Hospital of Infectious Diseases, Iasi, Romania; 18 Health Service Executive–Health Protection Surveillance Centre, Dublin, Ireland; 19 Lithuanian University of Health Sciences, Kaunas, Lithuania; 20 University Hospital Brno, Brno, Czechia; 21 Faculty of Medicine, Masaryk University, Brno, Czechia; 22 National Public Health Organisation (EODY), Athens, Greece; 23 Luxembourg Institute of Health, Luxembourg; 24 Robert Koch Institute, Berlin, Germany; 25 Servicio de Medicina Preventiva Hospital Universitario Son Espases, Servicio de Epidemiología, Consellería de Salut, Palma, Spain; 26 Teaching Public Health Institute of Split-Dalmatia County, Split, Croatia; 27 Carol Davila University of Medicine and Pharmacy, Bucharest, Romania; 28 Dr Victor Babes Clinical Hospital of Infectious and Tropical Diseases, Bucharest, Romania; 29 Centre Hospitalier de Luxembourg, Luxembourg; 30 Servicio de Epidemiología, Dirección General de Salud Pública, Consejería de Sanidad de Galicia, Santiago de Compostela, A Coruna, Spain; 31 Julius Center for Health Sciences and Primary Care, University Medical Center Utrecht, Utrecht, the Netherlands; 32 “Cantacuzino” National Military Medical Institute for Research-Development, Bucharest, Romania; 33 The members of these groups are listed under Collaborators

**Keywords:** SARS-CoV-2, Alpha, Delta, hospital, vaccine effectiveness, Europe

## Abstract

**Introduction:**

Two large multicentre European hospital networks have estimated vaccine effectiveness (VE) against COVID-19 since 2021.

**Aim:**

We aimed to measure VE against PCR-confirmed SARS-CoV-2 in hospitalised severe acute respiratory illness (SARI) patients ≥ 20 years, combining data from these networks during Alpha (March–June)- and Delta (June–December)-dominant periods, 2021.

**Methods:**

Forty-six participating hospitals across 14 countries follow a similar generic protocol using the test-negative case–control design. We defined complete primary series vaccination (PSV) as two doses of a two-dose or one of a single-dose vaccine ≥ 14 days before onset.

**Results:**

We included 1,087 cases (538 controls) and 1,669 cases (1,442 controls) in the Alpha- and Delta-dominant periods, respectively. During the Alpha period, VE against hospitalisation with SARS-CoV2 for complete Comirnaty PSV was 85% (95% CI: 69–92) overall and 75% (95% CI: 42–90) in those aged ≥ 80 years. During the Delta period, among SARI patients ≥ 20 years with symptom onset ≥ 150 days from last PSV dose, VE for complete Comirnaty PSV was 54% (95% CI: 18–74). Among those receiving Comirnaty PSV and mRNA booster (any product) ≥ 150 days after last PSV dose, VE was 91% (95% CI: 57–98). In time-since-vaccination analysis, complete all-product PSV VE was > 90% in those with their last dose < 90 days before onset; ≥ 70% in those 90–179 days before onset.

**Conclusions:**

Our results from this EU multi-country hospital setting showed that VE for complete PSV alone was higher in the Alpha- than the Delta-dominant period, and addition of a first booster dose during the latter period increased VE to over 90%.

**Key public health message**

**What did you want to address in this study?**

To understand how well the COVID-19 vaccine was performing in Europe against hospitalisation during SARS-CoV-2 Alpha and Delta variant periods, we present vaccine effectiveness results from a multi-country study of complete and booster dose COVID-19 vaccination among adults (aged 20 years and over).

**What have we learnt from this study?**

Between March and June 2021 (Alpha period), vaccine effectiveness against hospitalisation with laboratory-confirmed SARS-CoV-2 was 43% for partial vaccination and 86% for complete vaccination. For June to December 2021 (Delta period), vaccine effectiveness for complete vaccination was lower (52%) but with addition of an mRNA booster dose, effectiveness reached 91%, and remained > 90% up to 119 days after the booster dose.

**What are the implications of your findings for public health?**

In Europe in 2021, COVID-19 vaccine effectiveness results for the Alpha period indicated an excellent benefit for preventing hospitalisation after complete vaccination. During Delta variant circulation, however, a booster dose was required to achieve this level of effectiveness, and this was maintained for up to 4 months post booster.

## Introduction

COVID-19 has caused considerable morbidity and mortality in Europe since March 2020. International collaboration accelerated COVID-19 vaccine development and within the European Union (EU)/European Economic Area (EEA), by the end of December 2021, there were five COVID-19 vaccines authorised for use with conditional marketing [1,2]: two are spike protein-based mRNA vaccines: Comirnaty (BNT162b2; Pfizer-BioNTech) and Spikevax (mRNA-1273; Moderna); two are spike-protein-based adenoviral vector vaccines: Vaxzevria (ChAdOx1; AstraZeneca) and Jcovden (Ad26.COV 2.5; Johnson & Johnson); one is a subunit vaccine: Nuvaxovid (NVX-CoV2373; Novavax).

Earlier in 2021, the severe acute respiratory syndrome coronavirus 2 (SARS-CoV-2) Alpha variant was dominant in Europe, superseded by the Delta variant during the summer months. High efficacy was observed in randomised controlled trials of COVID-19 vaccines carried out before Alpha or Delta circulation [3-6]. However, evaluating the real-world COVID-19 vaccine performance is critical for understanding and evaluating vaccination programmes [7,8].

The I-MOVE (Influenza – Monitoring Vaccine Effectiveness in Europe) network expanded in 2020 to include monitoring of COVID-19 vaccine effectiveness (VE) and became the I-MOVE-COVID-19 network. The I-MOVE-COVID-19 and Vaccine Effectiveness, Burden and Impact Studies (VEBIS) networks in Europe have carried out hospital-based COVID-19 VE studies since 2021. Both networks use common generic protocols [9,10] and the test-negative case–control design.

We estimated VE against hospitalisation with PCR-confirmed SARS-CoV-2. In the absence of complete genetic sequencing information, we aimed to investigate VE during the Alpha and Delta periods between March and December 2021.

## Methods

### Setting and study period

**Figure 1:**
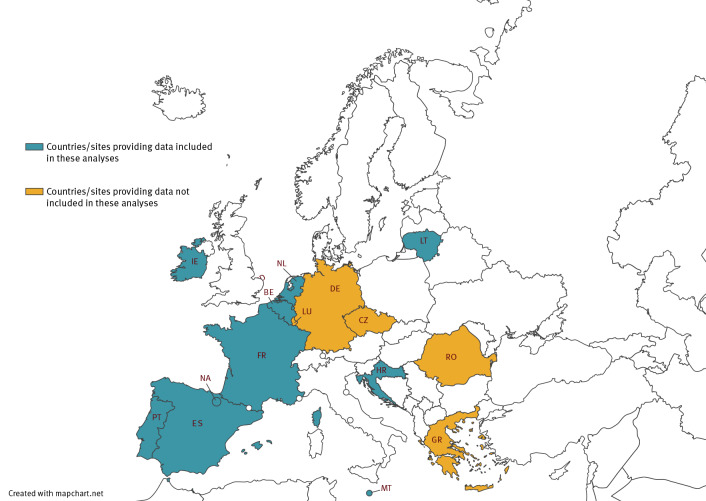
Countries and study sites participating in I-MOVE-COVID-19 and VEBIS hospital vaccine effectiveness studies, by provision of data for this analysis, Europe, 2021

I-MOVE: Influenza – Monitoring Vaccine Effectiveness in Europe; VEBIS: Vaccine Effectiveness, Burden and Impact Studies.

There were 15 I-MOVE-COVID-19 and VEBIS hospital VE study sites from 14 European countries participating in the two networks during the study period from 2 March to 25 December 2021 (Figure 1), including 46 hospitals.

Fifteen participating sites: Belgium (BE), Croatia (HR), Czechia (CZ), France (FR), Germany (DE), Greece (GR), Ireland (IE), Lithuania (LT), Luxembourg (LU), Malta (MT), the Netherlands (NL), Portugal (PT), Romania (RO), Spain 11 regions (ES), Spain Navarra region (NA). Ten included in this analysis: BE, ES, FR, HR, IE, LT, MT, NA, NL, PT.

We defined the Alpha- and Delta-dominant periods as weeks where ≥ 80% of variants sequenced were Alpha or Delta, respectively, in each country using GISAID data [11]. We included data only from these weeks: 3 March to 11 June for Alpha, and 21 June to 25 December for Delta dominance.

### Study design and case definitions

We used the test-negative case–control design [12], in which we defined cases as SARI patients testing SARS-CoV-2-positive by RT-PCR within 48 h of hospital admission or within the previous 14 days. Controls were SARI patients testing PCR-negative for SARS-CoV-2 within 48 h of admission. We used the ECDC definition for a possible COVID-19 case to define our study population of SARI patients (individuals hospitalised for at least 24 h, presenting with fever or cough or shortness of breath or sudden onset anosmia, ageusia, or dysgeusia) [13].

Study sites adapted their I-MOVE-COVID-19 or VEBIS VE network hospital study protocols [9,10] to their country-specific setting, as appropriate. Eleven sites included all SARI patients admitted to participating hospitals. The participating hospital in Belgium, the two in Lithuania and the over 20 hospitals in participating Spanish regions (except Navarra) included all SARI patients admitted on either 1 or 2 days each week. One site (the Netherlands) included a maximum of 10 SARI patients per week.

Study site teams collected demographic data (age, sex), clinical data (chronic conditions) and COVID-19 vaccination information via questionnaire, electronic medical records, vaccine registries or patient interview.

### Inclusion and exclusion criteria

We included patients aged 20 years and over who belonged to the age-specific target group for COVID-19 vaccination of their country at time of swab. We excluded patients with missing/erroneous key variables (age, sex, key dates, vaccination information). Study sites excluded patients with contraindications for vaccination.

### Definitions of vaccination status 

We defined SARI patients as partially vaccinated with primary series vaccination (PSV) 14 days after receiving one of two recommended doses of a two-dose vaccine; as completely vaccinated with PSV 14 days after receiving either the second of two recommended doses of a two-dose vaccine, or a single dose of JCovden; as having received a complete PSV plus booster dose 14 days after receiving the first mRNA booster; as unvaccinated if they did not receive any COVID-19 vaccine or were vaccinated with the first dose of a two-dose vaccine (or a single dose of JCovden) on or after the date of symptom onset. Those vaccinated with the second dose of a two-dose vaccine on or after their date of onset were recoded as partially vaccinated. Anyone vaccinated 1–14 days before onset was excluded. All analyses used unvaccinated as the comparison unexposed group (Table 1).

**Table 1:** Vaccination and analysis definitions for SARS-CoV-2 Alpha (3 March–11 June 2021) and Delta (21 June–25 December 2021) periods, I-MOVE-COVID-19 and VEBIS hospital vaccine effectiveness studies

Period	Alpha	Alpha, Delta	Delta	Delta					
Vaccination or group	Partial PSV	Complete PSV	Booster	Group A	Group B	Group C	Group D	Time since vaccination	Group E
**Definition**	14 days after receiving one of two recommended doses of a two-dose vaccine	14 days after receiving either: - the second of two recommended doses of a two-dose vaccine **or** - a single dose of JCovden	14 days after receiving the first mRNA booster	Complete PSV, with: - no booster, but eligible for a booster dose i.e. in the age-specified BDTG based on national recommendations - last PSV dose received ≥ 150 days before onset	Complete PSV + first mRNA booster in BDTG, with: - ≥ 150 days between the last PSV dose and the booster dose	Complete PSV only, for: - all in PSV target group regardless of time since last dose	Complete PSV only, for: - all in BDTG regardless of time since last dose	Complete PSV without and with booster for all vaccine products combined, for: - all in BDTG - by time since vaccination (30-, 60- and 90-day periods)	Complete PSV, in BDTG, with - symptom onset < 150 days from last PSV dose
**Analysis**	**Analysis 1:** Alpha period VE for partial and complete PSV		**Analysis 2:** Delta period VE for complete PSV ± booster, with last PSV dose ≥ 150 days before onset (complete PSV only), or ≥ 150 days before booster (complete PSV + booster)			**Analysis 3:** VE for complete PSV only for those in different vaccine target groups, regardless of time since last PSV dose		**Analysis 4:** VE for those in BDTG, complete PSV ± booster, over time	**Analysis 5:** VE for complete PSV only, with last PSV dose < 150 days before onset

BDTG: booster dose target group; I-MOVE: Influenza – Monitoring Vaccine Effectiveness in Europe; PSV: primary series vaccination; SARS-CoV-2: severe acute respiratory syndrome coronavirus 2; VE: vaccine effectiveness; VEBIS: Vaccine Effectiveness, Burden and Impact Studies.

For this analysis, we excluded those who had heterologous primary series doses or at least one unknown primary series product and we only included patients receiving the same product for both PSV doses (homologous PSV).

### Statistical analysis

We compared the odds of vaccination between cases and controls using logistic regression, calculating VE as 1 minus the OR of vaccination among cases and controls (expressed as a percentage). We included study site (as a fixed effect) and date of swab (modelled as a spline or categorical variable, with the best functional form designated by the Akaike information criterion) in all VE analyses.

We further adjusted the OR for age, sex and presence of at least one of the four commonly collected and COVID-19-relevant chronic conditions (asthma, diabetes, heart disease and lung disease).

For the age-specific analyses, we stratified the data into three age groups: 20–59, 60–79 and ≥ 80 years. We also performed stratified analyses by sex and by presence of at least one chronic condition (vs no chronic conditions). In time-since-vaccination analyses for the Delta period, we measured VE by days since last PSV dose (for those with complete PSV) and by days since mRNA booster dose (for those with complete PSV plus first booster), for 30-, 60- and 90-day periods.

During the Alpha period, we estimated VE against hospitalisation with COVID-19 in SARI patients for partial and complete PSV (Table 1; Analysis 1).

During the Delta period, we estimated VE against hospitalisation in SARI patients with COVID-19, restricting most analyses to patients eligible for a booster dose (i.e. who were in the age-specified booster dose target group based on national recommendations; Table 1). We estimated VE in all those with complete PSV without booster dose who had received their last PSV dose at least 150 days before onset (Group A; Analysis 2), and in those with a first mRNA booster, who had ≥ 150 days between their last PSV dose and the booster dose (Group B; Analysis 2). The 150-day restriction in Group A was used to facilitate comparison with Group B (on average, participating countries recommended first booster dose to be 5 months after last PSV dose). This analysis was conducted for the Delta period because the long duration of circulation resulted in longer delays from last PSV dose to onset.

In Analysis 3, we estimated VE for those receiving PSV only, in different vaccination target groups: the PSV target group (Group C), and the country-specific booster dose target group (BDTG; Group D). Time since last PSV was not taken into account (Table 1).

In Analysis 4, we estimated VE for complete PSV without and with booster by time since vaccination (using 30-, 60- and 90-day periods) for all vaccine products combined. 

In Analysis 5, we estimated VE for those with complete PSV in a country-specific BDTG with symptom onset less than 150 days from their last PSV dose (Table 1).

### Sensitivity analyses

We conducted sensitivity analyses excluding all SARI patients with known prior infection, and another restriction included only SARI patients with severe outcomes (either admission to an intensive care unit (ICU) or death in hospital). Where the number of cases or controls per parameter was < 10, a sensitivity analysis was conducted using Firth’s method of penalised logistic regression (PLR) to assess small sample bias [14,15], which we considered to be present if there was a difference of 10 percentage points between the PLR and original VE estimate. Any estimates meeting this criterion for small sample bias were not included, also we do not present any VE estimates in any stratum for which the total number of vaccinated cases and controls was < 20.

## Results

### Entire Alpha and Delta period (March to December 2021)

**Figure 2:**
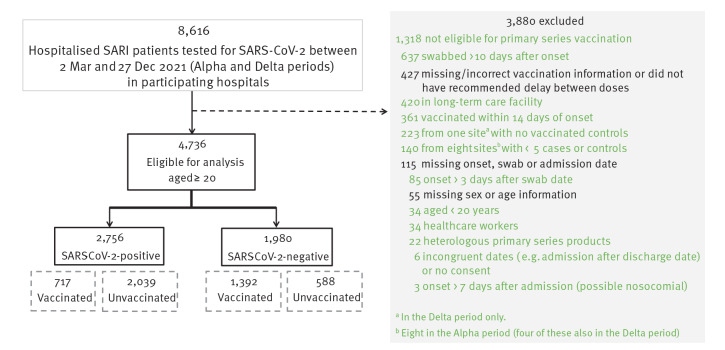
Exclusions for I-MOVE-COVID-19 and VEBIS hospital vaccine effectiveness studies, Europe, 2021 (n = 8,616)

I-MOVE: Influenza – Monitoring Vaccine Effectiveness in Europe; SARS-CoV-2: severe acute respiratory syndrome coronavirus 2; VEBIS: Vaccine Effectiveness, Burden and Impact Studies.

Fifteen sites submitted data on 8,616 SARI patients swabbed between 2 March and 25 December 2021 (Figure 1, Figure 2). Data from four sites were not eligible for inclusion in analysis from the outset, as their data included fewer than five cases (one site) or controls (two sites), or their participating hospitals had been designated ‘COVID only’ earlier in the pandemic and SARI patients were almost exclusively SARS-CoV-2-positive (one site). After exclusions, a further four sites were excluded from the Alpha period and one more from the Delta period (Figure 1) for having fewer than five cases or controls.

Included data are from 42 hospitals in 10 participating sites.

### Alpha period (3 March to 11 June 2021)

#### Descriptive analysis

Seven sites submitted data eligible for inclusion in this period (Table 2). After applying the study and analysis exclusion criteria (Figure 2), we included 1,087 cases and 538 controls who had been swabbed during the Alpha-dominant period. Most cases (n = 838; 77%) were younger than 80 years, while 320 controls (60%) were in this age group (Table 2). Fifty-seven per cent of cases (n = 622) and 54% of controls (n = 292) were male. Fifty-six per cent of cases (n = 606) and 74% of controls (n = 399) had one or more of the four commonly collected chronic conditions.

**Table 2:** Characteristic of cases and controls, I-MOVE-COVID-19 and VEBIS hospital vaccine effectiveness studies, Europe, SARS-CoV-2 Alpha and Delta periods March–December 2021 (n = 4,736)

Patient characteristic	Alpha period: January to June 2021 (n = 1,625)				Delta period: July to December 2021 (n = 3,111)			
	SARS-CoV-2 cases (n = 1,087)		Test-negative controls (n = 538)		SARS-CoV-2 cases (n = 1,669)		Test-negative controls (n = 1,442)	
	Number	%	Number	%	Number	%	Number	%
Median age (years)	69		76		63		72	
Age groups (years)								
20–59	247	22.7	69	12.8	695	41.6	348	24.1
60–79	591	54.4	251	46.7	644	38.6	641	44.5
≥ 80	249	22.9	218	40.5	330	19.8	453	31.4
Sex								
Male	622	57.2	292	54.3	984	59.0	815	56.5
Female	465	42.8	246	45.7	685	41.0	627	43.5
At least one chronic condition^a^								
No	481	44.3	139	25.8	929	55.7	449	31.1
Yes	606	55.7	399	74.2	740	44.3	993	68.9
COVID-19 vaccination status								
Unvaccinated	1,013	93.2	354	65.8	1,026	61.5	234	16.2
Partial vaccination only^b^	62	5.7	94	17.5	35	2.1	68	4.7
Complete PSV^c^	12	1.1	90	16.7	598	35.8	1,043	72.3
Complete PSV + first booster^d^	NA				10	0.6	97	6.7
Vaccine product among vaccinated: first dose								
Comirnaty	45	61.6	145	80.6	392	61.0	886	73.4
Vaxzevria	21	28.8	19	10.6	156	24.3	178	14.7
Spikevax	7	9.6	15	8.3	22	3.4	90	7.5
JCovden	0	0.0	1	0.6	72	11.2	47	3.9
Other/unknown^e^	1	NC	4	NC	1	NC	7	NC
Vaccine product among vaccinated: second dose								
Comirnaty	12	100	82	92.1	381	71.1	852	78.0
Vaxzevria	0	0	0	0.0	135	25.2	155	14.2
Spikevax	0	0	7	7.9	20	3.7	81	7.4
Other/unknown^e^	0	NC	0	NC	0	NC	5	NC
Vaccine product among vaccinated: first booster								
Comirnaty	NA				10	100	89	91.8
Spikevax					0	NC	7	7.2
Other/unknown^e^					0	NC	1	1.0
Severe outcomes								
Hospitalisation	1,087	100	538	100	1,669	100	1,442	100
ICU admission	231	21.7	20	4.0	330	20.0	119	8.4
No ICU admission	835	78.3	482	96.0	1,317	80.0	1,295	91.6
Missing^e^	21	NC	36	NC	22	NC	28	NC
In-hospital death	181	19.0	27	8.7	195	12.6	92	7.3
Discharged alive	744	78.2	242	77.5	1,286	83.0	1,082	85.9
Still in hospital/transferred	27	2.8	43	13.8	69	4.4	85	6.8
Missing^e^	135	NC	226	NC	119	NC	183	NC
Study site and country^f^								
Belgium	87	8.0	68	12.6	115	6.9	88	6.1
Czechia	NI				NI			
Spain	236	21.7	110	20.5	379	22.7	400	27.7
France	39	3.6	14	2.6	197	11.8	136	9.4
Germany	NI				NI			
Greece	NI				NI			
Croatia	579	53.3	74	13.8	433	25.9	92	6.4
Ireland	NI				75	4.5	53	3.7
Lithuania	24	2.2	6	1.1	34	2.0	22	1.5
Luxembourg	NI				NI			
Malta	NI				95	5.7	225	15.6
Navarra, Spain	37	3.4	85	15.8	42	2.5	109	7.6
the Netherlands	85	7.8	181	33.6	77	4.6	149	10.3
Portugal	NI				222	13.3	168	11.7
Romania	NI				NI			
**All sites**	**1,087**	100	**538**	100	**1,669**	100	**1,442**	100

ICU: intensive care unit; I-MOVE: Influenza – Monitoring Vaccine Effectiveness in Europe; NA: not applicable; NC: not calculated; NI: no data included; PSV: primary series vaccination; SARS-CoV-2: severe acute respiratory syndrome coronavirus 2; VEBIS: Vaccine Effectiveness, Burden and Impact Studies.

^a^ At least one of four commonly collected conditions (diabetes, heart disease, lung disease, asthma).

^b^ Partially vaccinated: any SARI patient who received only one of two recommended doses of a two-dose vaccine.

^c^ Completely vaccinated: any SARI patient who received either both of two recommended doses of a two-dose vaccine, or a single dose of Jcovden.

^d^ Vaccinated with first booster: any SARI patient who received either both of two recommended doses of a two-dose vaccine, or a single dose of Jcovden, plus one booster dose.

^e^ Missing totals not included in percentages.

^f^ Eight sites submitting data during the Alpha and five during the Delta period did not have sufficient eligible SARI patients for inclusion in these analyses (this table gives final numbers by site, after exclusions).

**Figure 3:**
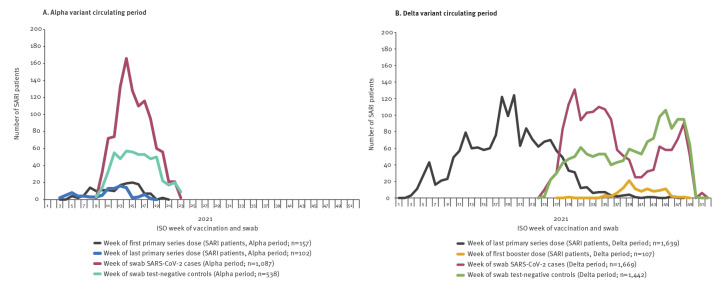
Number of SARI patients by case status and week of COVID-19 vaccination (second and booster doses) or swab, I-MOVE-COVID-19 and VEBIS hospital vaccine effectiveness studies, Europe, 2021 (n = 4,736)

I-MOVE: Influenza – Monitoring Vaccine Effectiveness in Europe; SARI: severe acute respiratory infection; SARS-CoV-2: severe acute respiratory syndrome coronavirus 2; VEBIS: Vaccine Effectiveness, Burden and Impact Studies.

There were 62 (6%) partially and 12 (1%) completely vaccinated cases, 94 (18%) partially and 90 (17%) completely vaccinated controls. Vaccination rollout continued as cases and controls were selected (Figure 3).

All 12 completely vaccinated cases had received Comirnaty. Among the 90 completely vaccinated controls, 82 (91%) had received two doses of Comirnaty, seven (8%) had received Spikevax and one (< 1%) had received a single dose of JCovden (Table 2).

A higher proportion of cases than controls were admitted to ICU or died in hospital (22% vs 4% and 19% vs 9%, respectively; Table 1). Most (> 90%) cases and controls were swabbed between weeks 10–18 (Figure 3).

#### Vaccine effectiveness estimates

##### Analysis 1: Vaccine effectiveness in patients ≥ 20 years for partial and complete primary series vaccination

Partial vaccination VE among those aged ≥ 20 years (all vaccine products combined) was 43% (95% confidence interval (CI): 13–62); 45% (95% CI: 8–67) for Comirnaty and 31% (95% CI: −46 to 67) for Vaxzevria. Sensitivity analyses excluding SARI patients with known prior infection are appended in Supplementary Table S1, where the VE was within 2 percentage points for partial VE. For SARI patients with severe outcomes during the Alpha period, numbers were too small to estimate VE.

Complete PSV (all products) VE during this period was 86% (95% CI: 71–93); 85% (95% CI: 69–92) for Comirnaty (Table 3). There was insufficient sample size to estimate complete PSV VE for any other vaccine product during this period. The point estimate of VE for sensitivity analyses (prior infection) were identical for complete PSV; these results are made available in Supplementary Table S1. Again, there was not sufficient sample size for the sensitivity analysis for severe outcomes.

**Table 3:** Effectiveness of COVID-19 partial and complete primary series vaccination against hospitalisation among adults (≥ 20 years) with complete primary vaccination, by dose, age group and vaccine product, I-MOVE-COVID-19 and VEBIS hospital vaccine effectiveness studies, Europe, Alpha and Delta periods March–December 2021 (n = 4,736)

PSV vaccine product	Vaccinated/unvaccinated cases; vaccinated /unvaccinated controls		VE^a^ (95% CI)	
Analysis 1: VE of partial and complete PSV, Alpha period (March–June 2021; n = 1,625)				
	Partial PSV	Complete PSV	Partial PSV	Complete PSV
**All products combined**	**7 sites^b^; n = 1,523^c^**	**7 sites^b^; n = 1,469^d^**		
All ≥ 20 years	62/1,013; 94/354	12/1,013; 90/354	43 (13 to 62)	86 (71 to 93)
**Comirnaty PSV**	**7 sites^b^; n = 1,463**	**6 sites^e^; n = 1,434**		
All ≥ 20 years	33/1,013; 63/354	12/991; 82/349	45 (8 to 67)	85 (69 to 92)
**Vaxzevria PSV**	**6 sites^e^; n = 1,358**	**0 sites^f^; n = 0**		
All ≥ 20	21/975; 19/343	NA	31 (−46 to 67)	NA
Analysis 2: VE in patients eligible for a booster dose. Group A: those who received complete PSV only, with last PSV dose administered ≥ 150 days before onset. Group B: those who received PSV plus an mRNA booster dose administered ≥ 150 days after last PSV dose. Delta period (June–December 2021; n = 3,111)				
	Complete PSV (Group A)	Complete PSV + mRNA booster (Group B)	Complete PSV (Group A)	Complete PSV + mRNA booster (Group B)
**All products combined**	**7 sites^g^; n = 524^h^**	**2 sites^i^; n = 88^j^**		
All ≥ 20 years	103/102; 268/51	4/39; 21/24	52 (18 to 71)	91 (57 to 98)
**Comirnaty PSV**	**6 sites^k^; n = 435**	**2 sites^l^; n = 88**		
All ≥ 20 years	80/75; 233/47	4/39; 21/24	54 (18 to 74)	91 (57 to 98)
**Vaxzevria PSV**	**2 sites^l^; n = 107**	**0 sites^m^**		
All ≥ 20 years	6/65; 2/34	ND	ND^n^	NA
**JCovden PSV**	**3 sites^l^; n = 141**	**0 sites^m^**		
All ≥ 20 years	5/92; 6/38	ND	ND^n^	NA
**Spikevax PSV**	**2 sites^m^; n = 112**	**0 sites^m^**		
All ≥ 20 years	4/65; 9/34	ND	ND^n^	NA
Analysis 3: VE in patients vaccinated ≥ 14 days before onset for those with PSV only, for those in the PSTG (Group C) and those in the BDTG (Group D); Delta period (June–December 2021; n = 3,111)				
	Complete PSV, in PSTG (Group C)	Complete PSV, in BDTG (Group D)	Complete PSV, in PSTG (Group C)	Complete PSV, in BDTG (Group D)
**All products combined**	**10 sites^o^; n = 2,901^p^**	**7 sites^g^; n = 735^q^**		
All ≥ 20 years	598/1,026; 1,043/234	168/102; 414/51	79 (74 to 83)	69 (51 to 80)
20–59 years	124/558; 223/92	21/45; 57/14	86 (80 to 91)	86 (62 to 95)
60–79 years	271/350; 479/92	87/41; 193/24	80 (72 to 85)	71 (44 to 85)
≥ 80 years	203/118; 341/50	60/16; 164/13	38 (−4 to 62)	40 (−64 to 78)
No chronic condition^r^	209/701; 286/107	39/48; 78/13	86 (80 to 90)	79 (47 to 91)
Any chronic condition^r^	389/325; 757/127	129/54; 336/38	71 (61 to 78)	65 (40 to 79)
**Comirnaty PSV**	**10 sites^o^; n = 2,390**	**7 sites^g^; n = 560**		
All ≥ 20 years	371/1,026; 759/234	96/102; 311/51	82 (77 to 86)	74 (57 to 84)
20–59 years	57/558; 140/92	9/45; 35/14	91 (86 to 94)	92 (73 to 98)
60–79 years	140/350; 310/92	38/41; 127/24	82 (74 to 88)	79 (54 to 90)
≥ 80 years	174/118; 309/50	49/16; 149/13	49 (11 to 71)	56 (−31 to 85)
No chronic condition^r^	37/661; 16/98	6/43; 2/7	55 (7 to 78)	ND^n^
Any chronic condition^r^	263/325; 565/127	81/54; 256/38	74 (64 to 81)	69 (46 to 82)
**Vaxzevria PSV**	**10 sites^o^; n = 1,548**	**3 sites^s^; n = 203**		
All ≥ 20 years	135/1,026; 153/234	36/92; 37/38	69 (57 to 78)	50 (−4 to 75)
20–59 years	31/558; 29/92	4/44; 2/12	59 (18 to 79)	ND^n^
60–79 years	85/350; 118/92	26/36; 34/18	81 (69 to 88)	63 (10 to 85)
≥ 80 years	19/118; 6/50	6/12; 1/8	11 (−183 to 72)	ND^n^
No chronic condition^r^	50/701; 53/107	12/43; 9/7	79 (65 to 88)	74 (−14 to 94)
Any chronic condition^r^	85/325; 100/127	24/49; 28/31	63 (43 to 76)	37 (−38 to 72)
**JCovden PSV**	**8 sites^t^; n = 1,309**	**3 sites^s^; n = 162**		
All ≥ 20 years	65/976; 47/221	16/92; 16/38	60 (37 to 75)	60 (1 to 84)
20–59 years	30/525; 26/84	5/44; 7/12	79 (58 to 90)	ND^n^
60–79 years	30/335; 14/88	8/36; 6/18	21 (−76 to 65)	
≥ 80 years	5/116; 7/49	3/12; 3/8	ND^n^	
No chronic condition^r^	37/661; 16/98	6/43; 2/7	55 (7 to 78)	
Any chronic condition^r^	28/315; 31/123	10/49; 14/31	60 (26 to 78)	64 (−4 to 87)
**Spikevax PSV**	**5 sites^u^; n = 1,117**	**1 site^v^; n = 53**		
All ≥ 20 years	20/849; 61/187	ND*^w^*	89 (81 to 94)	ND^w^
20–59 years	3/439; 21/60		97 (90 to 99)	
60–79 years	12/306; 28/80		82 (60 to 72)	
≥ 80 years	5/104; 12/47		ND^n^	
No chronic condition^r^	8/566; 19/75		94 (84 to 98)	
Any chronic condition^r^	12/283; 42/112		83 (64 to 92)	
Analysis 4: Time-since-vaccination analysis. VE in patients vaccinated ≥ 14 days before onset who are in a BDTG, with complete PSV and those with complete PSV plus mRNA booster. Delta period (June–December 2021; n = 3,111)				
	Complete PSV only	Complete PSV + mRNA booster	Complete PSV only	Complete PSV + mRNA booster
**All products combined**	**7 sites^g^; n = 735^q^**	**3 sites^s^; n = 168^j^**		
All ≥ 20 years	168/102; 414/51	4/92; 34/38	69 (52 to 80)	94 (77 to 98)
Time since last dose (days)				
14–29	0/102; 6/51	1/92; 16/38	ND^n^	ND^n^
30–59	1/102; 7/51	3/92; 12/38	ND^n^	ND^n^
≥ 60	167/102; 401/51	0/92; 6/38	68 (50 to 80)	ND^n^
14–59	1/102; 13/51	4/92; 28/38	ND^n^	92 (67 to 98)
60–119	27/102; 55/51	0/92; 6/38	78 (55 to 90)	ND^n^
≥ 120	140/102; 346/51	0/92; 0/38	66 (45 to 78)	ND^n^
14–89	6/102; 35/51	4/92; 33/38	93 (76 to 98)	93 (75 to 98)
90–119	22/102; 33/51	0/92; 1/38	73 (37 to 88)	ND^n^
14–119	28/102; 68/51	4/92; 34/38	81 (62 to 91)	94 (76 to 98)
120–149	37/102; 78/51	0/92; 0/38	73 (48 to 86)	ND^n^
150–179	30/102; 102/51	0/92; 0/38	70 (43 to 85)	ND^n^
≥ 180	73/102; 166/51	0/92; 0/38	25 (−44 to 61)	ND^n^
Analysis 5: VE of complete PSV among patients without booster dose but in BDTG and vaccinated 14–149 days before onset; Delta period (June–December 2021; n = 3,111)				
**All products combined**	**Three sites^s^; n = 308^x^**			
All ≥ 20	53/92; 125/38		76 (57 to 87)	
20–59 years	9/44; 40/12		92 (75 to 98)	
60–79 years	34/36; 73/18		70 (31 to 87)	
80 years	10/12; 12/8		ND^y^	
No chronic condition^r^	17/43; 21/7		77 (24 to 93)	
Any chronic condition^r^	36/49; 104/31		76 (54 to 88)	

BDTG: booster dose target group; CI: confidence interval; ICU: intensive care unit; I-MOVE: Influenza – Monitoring Vaccine Effectiveness in Europe; NA: not applicable; ND: not done; PSTG: primary series target group; PSV: primary series vaccination; VE: vaccine effectiveness; VEBIS: Vaccine Effectiveness, Burden and Impact Studies.

^a^ Odds ratio adjusted by study site as a fixed effect, time (restricted cubic spline of swab date, or swab month as a categorical term, depending on model), age or 5-year age group (linear or categorical term depending on model), sex, and presence of at least one of four chronic conditions (asthma, diabetes, heart disease, lung disease).

^b^ Seven study sites: Belgium, Croatia, France, Lithuania, Navarra, the Netherlands and Spain.

^c^ n = 1,523 after dropping 102 records from patients with complete vaccination.

^d^ n = 1,469 after dropping 156 records from patients with partial vaccination.

^e^ Six study sites: Belgium, Croatia, France, Navarra, the Netherlands and Spain.

^f^ None of the participating sites with patients receiving complete vaccination had used Vaxzevria during the Alpha-dominant period.

^g^ Seven study sites: Belgium, France, Malta, Navarra, the Netherlands, Portugal and Spain.

^h^ n = 524 after dropping 2,195 records from patients who were not yet in the target group for booster dose vaccination, 211 from those vaccinated < 150 days before onset, 101 from those with a booster dose, 213 from patients with partial vaccination, and 59 from sites with fewer than five 5 cases/controls (or a total of cases and controls < 20).

^i^ Two study sites: Belgium and Spain.

^j^ n = 88 after dropping 2,195 records from patients who were not yet in the target group for booster dose vaccination, 634 from those with partial vaccination or complete vaccination without booster, 114 from sites with fewer than five cases/controls (or a total of cases and controls < 20) and 80 from one site with no vaccinated cases.

^k^ Six study sites: France, Malta, Navarra, the Netherlands, Portugal and Spain.

^l^ Two study sites: France and Spain.

^m^ None of the participating sites recruited patients who had received a booster dose after having a complete primary course of Vaxzevria, JCovden or Spikevax.

^n^ Numbers are too small to provide robust VE estimates when there are ≤ 20 vaccinated cases and controls in total.

^o^ Ten study sites: Belgium, Croatia, France, Ireland, Lithuania, Malta, Navarra, the Netherlands, Portugal and Spain.

^p^ n = 2,901 after dropping 107 from those with a booster dose and 103 from those with partial vaccination only.

^q^ n = 735 after dropping 2,195 records from patients who were not yet in the target group for booster dose vaccination, 101 from those with a booster dose, 21 from patients with partial vaccination, and 59 from sites with fewer than five cases or controls (or a total of cases and controls < 20).

^r^ In this analysis stratified by chronic condition, the adjustment for presence of at least one chronic condition was removed.

^s^ Three study sites: Belgium, France and Spain.

^t^ Eight study sites: Belgium, Croatia, France, Malta, Navarra, the Netherlands, Portugal and Spain.

^u^ Five study sites: Croatia, France, the Netherlands, Portugal and Spain.

^v^ One study site: Spain.

^w^ Pooled analysis not possible with just one site.

^x^ n = 308 after dropping 2,195 records from patients who were not yet in the target group for booster dose vaccination, 371 from those who were vaccinated ≥ 150 days before onset, 115 from sites with fewer than five cases/controls (or a total of cases and controls < 20), 101 from patients with a booster dose, and 21 from those with partial vaccination only.

^y^ Evidence of small sample bias so VE estimate not shown.

Partial VE was < 60% in those aged 60–79 years and < 30% in those ≥ 80 years; there was only sufficient sample size to estimate complete PSV VE in the oldest age group (≥ 75%); for partial and complete PSV results among adults (≥20 years) in the PSV target group, overall and in those eligible for a booster dose, see Supplementary Table S2. During this period, few individuals aged 20–59 years had been targeted for vaccination, so we could not provide estimates for this age group.

### Delta period (21 June–25 December 2021)

#### Descriptive analysis

Ten sites submitted data eligible for inclusion in this period (Table 2). After applying the study and analysis exclusion criteria (Figure 2), we included 1,669 cases and 1,442 controls. Most cases (n = 1,339; 80%) were aged < 80 years, while 989 controls (69%) were in this age group (Table 1). Fifty-nine per cent of cases (n = 984) and 57% of controls (n = 815) were male. Forty-four per cent of cases (n = 740) and 69% of controls (n = 993) had one or more of the four commonly collected chronic conditions.

There were 35 (2%) partially and 598 (36%) completely vaccinated cases, 68 (5%) partially and 1,043 (72%) completely vaccinated controls (Table 2). Ten cases (1%) and 97 controls (7%) had received a first booster. Vaccination rollout continued as cases and controls were selected (Figure 3).

Of the 598 completely vaccinated cases, 391 (65%) had received an mRNA vaccine product (371 Comirnaty and 20 Spikevax), 135 had received Vaxzevria (23%) and 72 (12%) had received JCovden. Among the 1,043 completely vaccinated controls, 838 (80%) had received an mRNA product (759 Comirnaty and 79 Spikevax), 153 (15%) had received Vaxzevria and 47 (5%) had received JCovden (Table 2).

A higher proportion of cases than controls were admitted to ICU or died in hospital (20% vs 8% and 13% vs 7%; Table 2). Most cases (n = 1,095; 66%) were swabbed in weeks 28–39, while most controls (n = 895; 62%) were swabbed in weeks 38–49 (Figure 3).

#### Vaccine effectiveness estimates

##### Analysis 2: Vaccine effectiveness in patients ≥ 20 years and eligible for a booster dose

The VE for complete PSV alone (all products combined) was 52% (95% CI: 18–71); 91% (95% CI: 57–98) in those with complete PSV and an mRNA booster dose. For Comirnaty, these estimates were 54% (95% CI: 18–74) and 91% (95% CI: 57–98), respectively. Insufficient sample size prevented estimation of VE for other vaccine products (Table 3). The VE in sensitivity analysis (severe outcomes) was 32 percentage points higher for complete PSV; small sample size precluded sensitivity analysis for those with booster dose. Excluding those with known prior infection gave VE within 4 percentage points of this main analysis for those with booster; complete sensitivity analysis data are accessible in Supplementary Table S1.

##### Analysis 3: Vaccine effectiveness for complete primary series vaccination only, for those in different vaccination target groups

For SARI patients in Group C (PSV target group), complete PSV VE was 79% (95% CI: 74–83) for all products combined and 82% (95% CI: 77–86) for Comirnaty. For Group D (patients in BDTG), complete PSV was 69% (95% CI: 51–80) for all products and 74% (95% CI: 57–84) for Comirnaty. Sample size was small and confidence intervals wide for other products, particularly for stratified estimates (Table 3).

##### Analysis 4: Vaccine effectiveness by time since vaccination for those in a booster dose target group

There was insufficient sample size to estimate VE for complete PSV only in those with onset < 60 days since vaccination, and in those who had received a first mRNA booster dose for any period ≥ 120 days since last booster dose.

For complete PSV without booster, among those who received their last PSV dose 60–119 and ≥ 120 days before onset, VE was 79% (95% CI: 57–90) and 66% (95% CI: 45–78), respectively. The VE in this group was highest in those receiving their last PSV dose 14–89 days before onset, at 93% (95%CI: 77–98). For those with onset between 90 and 179 days after their last PSV dose, VE remained at or above 70%, but dropped to 33% (95% CI: −27 to 64) in those with last PSV dose ≥ 180 days before onset. Among those with a first mRNA booster, VE was 92% (95% CI: 67–98) if the booster dose was received 14–59 days before onset and remained > 90% up to 119 days. The VE in sensitivity analysis (severe outcomes) was 11 percentage points higher for complete PSV among those vaccinated ≥ 14 days before onset; the full sensitivity analysis is accessible in Supplementary Table S1.

##### Analysis 5: Vaccine effectiveness for complete primary series vaccination for those aged ≥ 20 years in a booster dose target group (but without booster dose) with last primary series vaccination dose < 150 days before onset

Complete PSV VE was 76% (95% CI: 57–87). Age stratification showed the highest VE in the youngest age group (20–59 years), at 92% (95% CI: 75–98) (Table 3).

## Discussion

In our study, during the Alpha period, Comirnaty VE against COVID-19 hospitalisation in adults aged ≥ 20 years was higher for complete PSV (86%) than for partial vaccination (43%). During Delta-dominant circulation, for SARI patients with their last PSV or mRNA booster dose at least 150 days before onset, VE for complete PSV was 52%, rising to 91% on addition of the mRNA booster dose. For those having mRNA booster dose administered within 120 days before onset of symptoms, VE was at least 90%. Sample size precluded any booster dose VE estimates after this time. 

Looking at time since vaccination for those with complete PSV only, VE was 93% for those with last dose within 90 days of onset, at least 70% for those with last dose 90–179 days before onset, and 25% for those with their last dose ≥ 180 days before onset. This may indicate that the most important criterion for effectiveness is the time which has elapsed since receipt of last dose of any vaccine, regardless of number of doses (we observed a VE of 93% in those with complete PSV alone and in those with mRNA booster, when last dose was received 14–89 days before onset). However, in the longer period 14–119 days from last dose to onset, we observed higher VE in those with booster dose (94% vs 81%). More analyses with greater sample size need to be carried out to investigate this further. Importantly, we observed during the Delta period that VE in patients < 80 years remained over 60% in those without a booster dose 150 days from their last PSV dose, and was over 50% in the oldest age group, with the median time since vaccination being considerably shorter in the younger vs the older age groups (176 vs 195 days, respectively). In addition, albeit with overlapping confidence intervals, we observed a ca 40 percentage point greater VE in those with chronic conditions in a BDTG on receipt of a booster dose.

Our study had some important limitations. We sought to mitigate the heterogeneity inherent in a multi-country observational study by the common use of a generic protocol in all sites and the adjustment of VE estimates by site. In addition, the test-negative design should reduce heterogeneity between study sites; conducted in a hospital setting, this design should limit the presence of any healthcare-seeking bias.

The ECDC case definition for possible COVID-19 patients [13] was developed to respond to the broader symptom range of COVID-19 and includes a wider range of symptoms than those used in the World Health Organization SARI case definition [16]. Using this more sensitive SARI case definition could have resulted in the inclusion of more patients hospitalised with, rather than because of, COVID-19; i.e. potentially vaccinated patients with milder COVID-19, hospitalised for another cause. This concern was highlighted during circulation of the milder Omicron variant [17,18]; it would result in a higher proportion of included vaccinated cases, underestimating the VE. In sensitivity analyses using more specific severe outcomes (ICU admission or death), we found considerably higher VE in those with complete PSV during the Delta, but not during the Alpha period.

Prior SARS-CoV-2 infection can provide some immunity to unvaccinated SARI patients. In some participating countries in our study (e.g. France, Spain), known prior infection resulted in a delay of the next scheduled dose. In other countries, some individuals may also have delayed vaccination if they had a known infection. This could result in milder disease in unvaccinated cases being discovered when a SARI patient is hospitalised with (not because of) COVID-19, as described above. In sensitivity analyses estimating VE after excluding those with prior infection, we found almost identical VE estimates, whether for partial and complete PSV (Alpha) or those with and without booster (Delta), although this information was missing in almost one-third of patients (data not shown).

Earlier in 2021, the SARS-CoV-2 Alpha variant was dominant in Europe, superseded by the Delta variant during the summer months. In participating countries, vaccine rollout was initially limited to vulnerable populations [19] (e.g. older adults, those in long-term care or those with co-morbidities; for a list of target groups for primary course and first booster dose vaccination by participating countries, see Supplementary Tables S3 and S4) with more susceptibility to COVID-19. In particular, the Alpha-dominant circulating period coincided with the early phase of the vaccine roll-out in the EU/EEA. This was based on an age-staggered approach that prioritised older adults and those at risk of severe outcome, as well as healthcare workers. Therefore, recruitment of study participants in this period would have mainly been among those initial target groups, and we could not provide VE estimates in all age groups during the Alpha-dominant period. Similarly, estimates could only be calculated for partial PSV for one other product than Comirnaty (Vaxzevria), while complete PSV VE could only be provided for Comirnaty, as this was the main COVID-19 vaccine product distributed in the EU/EEA at this time [20].

Despite these limitations, our all-product, complete PSV VE against COVID-19 hospitalisation in those aged at least 20 years during the Alpha-dominant period (86%; 95%CI: 71–93) was similar to or slightly lower than some other published estimates. For example, studies in those aged ≥ 18 years showed VE of 87% (95% CI: 81–91) in the United States (US) [21] and 93% (95% CI: 89–96) in a systematic review [22]. Other studies in Canada, the United Kingdom (UK), Israel and Denmark have shown somewhat higher estimates during this period [23-26]. Comparisons across studies should bear in mind differences in study design, minimal hospitalisation time, age groups (≥ 16 or ≥ 18 years vs our ≥ 20 years), potentially different severity criteria, and pooling of results for different severe outcomes (e.g. combining hospitalisation and death).

Similar to our findings, other studies have reported Comirnaty VE estimates for partial PSV to be lower than for complete PSV, and VE in the oldest age group to be lower than in younger age groups [25,27,28] during the Alpha period.

Our PSV VE estimates during the Delta-dominant circulating period (from 52% to 79% overall) were slightly lower than those published elsewhere (by product, overall and by age group). For example, other studies have reported PSV VE estimates against COVID-19 hospitalisation of 67–99% (Norway; combined vaccine products; cohort study [29]), 74–84% (Hungary; Comirnaty, Spikevax and Vaxzevria; cohort study [30]), 75–80% (US; Comirnaty; cohort study [31]), ≥ 95% (Canada; Comirnaty, Spikevax and Vaxzevria; test-negative design [32]) and ≥ 96% (UK; Comirnaty; test-negative design [24,26]). This could be due to our more sensitive SARI case definition, described above, as well as variations in time since vaccination between studies.

It is well documented that the VE against COVID-19 hospitalisation, including groups at higher risk for severe disease, declines with increasing time since vaccination [29,33-35], particularly in older adults [34,35]. A systematic review indicated that, as in our study, other studies had only small (9–10 percentage points) changes in VE ≤ 180 days from last primary series dose, with VE remaining ≥ 70% up to this time [35]. The temporal evolution of VE varies by age, vaccine product, circulating virus variant and region. This highlights the importance of multi-country studies using a similar generic protocol in an attempt to standardise the methodological approach and provide robust pooled results.

## Conclusions

Our results from an EU multi-country hospital setting add to the evidence already available from other settings. Our study indicates that the VE point estimate of complete PSV was higher during the period of Alpha- than during Delta-dominant circulation (overall and for those vaccinated 14–89 days since last PSV dose), and that addition of a first booster dose during Delta-dominant circulation increased VE to over 90%. Finally, during the Delta-dominant period, we observed declining effectiveness over time, likely due to waning immunity. Despite this, VE was 93% 14–89 days after the last booster or PSV dose and remained 70% or greater 90–179 days from last PSV dose (in those without booster).

## Supplementary Data

379000 Supplement

## References

[1] Harder T Koch J Vygen-Bonnet S Külper-Schiek W Pilic A Reda S Efficacy and effectiveness of COVID-19 vaccines against SARS-CoV-2 infection: interim results of a living systematic review, 1 January to 14 May 2021. Euro Surveill. 2021;26(28):2100563. 10.2807/1560-7917.ES.2021.26.28.2100563 34269175 PMC8284046

[2] European Medicines Agency (EMA). COVID-19 medicines. Amsterdam: EMA. [Accessed: 28 Mar 2021]. Available from: https://www.ema.europa.eu/en/human-regulatory/overview/public-health-threats/coronavirus-disease-covid-19/treatments-vaccines/treatments-vaccines-covid-19-authorised-medicines

[3] Baden LR El Sahly HM Essink B Kotloff K Frey S Novak R Efficacy and safety of the mRNA-1273 SARS-CoV-2 vaccine. N Engl J Med. 2021;384(5):403-16. 10.1056/NEJMoa2035389 33378609 PMC7787219

[4] Polack FP Thomas SJ Kitchin N Absalon J Gurtman A Lockhart S Safety and efficacy of the BNT162b2 mRNA Covid-19 vaccine. N Engl J Med. 2020;383(27):2603-15. 10.1056/NEJMoa2034577 33301246 PMC7745181

[5] Sadoff J Gray G Vandebosch A Cárdenas V Shukarev G Grinsztejn B Safety and efficacy of single-dose Ad26.COV2.S vaccine against Covid-19. N Engl J Med. 2021;384(23):2187-201. 10.1056/NEJMoa2101544 33882225 PMC8220996

[6] Voysey M Clemens SAC Madhi SA Weckx LY Folegatti PM Aley PK Safety and efficacy of the ChAdOx1 nCoV-19 vaccine (AZD1222) against SARS-CoV-2: an interim analysis of four randomised controlled trials in Brazil, South Africa, and the UK. Lancet. 2021;397(10269):99-111. 10.1016/S0140-6736(20)32661-1 33306989 PMC7723445

[7] World Health Organization WHO). Evaluation of COVID-19 vaccine effectiveness. Geneva: WHO; 2021. Available from: https://www.who.int/publications-detail-redirect/WHO-2019-nCoV-vaccine_effectiveness-measurement-2021.1

[8] Tregoning JS Flight KE Higham SL Wang Z Pierce BF . Progress of the COVID-19 vaccine effort: viruses, vaccines and variants versus efficacy, effectiveness and escape. Nat Rev Immunol. 2021;21(10):626-36. 10.1038/s41577-021-00592-1 34373623 PMC8351583

[9] Epiconcept. European study of COVID-19 vaccine effectiveness against hospitalised SARI patients laboratory-confirmed with SARS-CoV-2. Draft generic protocol. Paris: Epiconcept; 2021. Available from: https://www.imoveflu.org/wp-content/uploads/2021/03/08feb2021_draft_generic_VE_protocol_hospital-based_COVID-19_v07.pdf

[10] European Centre for Disease Prevention and Control (ECDC). Core protocol for ECDC studies of COVID-19 vaccine effectiveness against hospitalisation with Severe Acute Respiratory Infection laboratory-confirmed with SARS-CoV-2, version 1.0. Stockholm: ECDC; 2021. Available from: https://www.ecdc.europa.eu/en/publications-data/core-protocol-ecdc-studies-covid-19-vaccine-effectiveness-against-hospitalisation

[11] Shu Y McCauley J . GISAID: Global initiative on sharing all influenza data - from vision to reality. Euro Surveill. 2017;22(13):30494. 10.2807/1560-7917.ES.2017.22.13.30494 28382917 PMC5388101

[12] Jackson ML Nelson JC . The test-negative design for estimating influenza vaccine effectiveness. Vaccine. 2013;31(17):2165-8. 10.1016/j.vaccine.2013.02.053 23499601

[13] Peralta-Santos A. Assessment of COVID-19 surveillance case definitions and data reporting in the European Union. Briefing requested by the ENVI committee. Brussels: European Parliament; July 2020. Available from: https://www.europarl.europa.eu/RegData/etudes/BRIE/2020/652725/IPOL_BRI(2020)652725_EN.pdf

[14] Peduzzi P Concato J Feinstein AR Holford TR . Importance of events per independent variable in proportional hazards regression analysis. II. Accuracy and precision of regression estimates. J Clin Epidemiol. 1995;48(12):1503-10. 10.1016/0895-4356(95)00048-8 8543964

[15] Covenay J. FIRTHLOGIT: Stata module to calculate bias reduction in logistic regression. Boston: Boston College Department of Economics; 2008. [Accessed: 3 Feb 2020]. Available from: https://econpapers.repec.org/software/bocbocode/s456948.htm

[16] World Health Organization (WHO). WHO surveillance case definitions for ILI and SARI. Geneva: WHO; 2014. Available from: https://www.who.int/teams/global-influenza-programme/surveillance-and-monitoring/case-definitions-for-ili-and-sari

[17] Feikin DR Abu-Raddad LJ Andrews N Davies MA Higdon MM Orenstein WA Assessing vaccine effectiveness against severe COVID-19 disease caused by omicron variant. Report from a meeting of the World Health Organization. Vaccine. 2022;40(26):3516-27. 10.1016/j.vaccine.2022.04.069 35595662 PMC9058052

[18] Stowe J Andrews N Kirsebom F Ramsay M Bernal JL . Effectiveness of COVID-19 vaccines against Omicron and Delta hospitalisation, a test negative case-control study. Nat Commun. 2022;13(1):5736. 10.1038/s41467-022-33378-7 36180428 PMC9523190

[19] European Centre for Disease Prevention and Control (ECDC). Overview of the implementation of COVID-19 vaccination strategies and vaccine deployment plans in the EU/EEA. Stockholm: ECDC; 2021 [Accessed: 28 Mar 2021]. Available from: https://www.ecdc.europa.eu/en/publications-data/overview-implementation-covid-19-vaccination-strategies-and-vaccine-deployment

[20] European Centre for Disease Prevention and Control (ECDC). COVID-19 vaccine tracker. Stockholm: ECDC. [Accessed: 28 Mar 2021]. Available from: https://vaccinetracker.ecdc.europa.eu/public/extensions/COVID-19/vaccine-tracker.html#uptake-tab

[21] Tenforde MW Patel MM Ginde AA Douin DJ Talbot HK Casey JD . Effectiveness of severe acute respiratory syndrome coronavirus 2 messenger rna vaccines for preventing coronavirus disease 2019 hospitalizations in the United States. Clin Infect Dis. 2022;74(9):1515-24. 10.1093/cid/ciab687 34358310 PMC8436392

[22] Liu Q Qin C Liu M Liu J . Effectiveness and safety of SARS-CoV-2 vaccine in real-world studies: a systematic review and meta-analysis. Infect Dis Poverty. 2021;10(1):132. 10.1186/s40249-021-00915-3 34776011 PMC8590867

[23] Chung H He S Nasreen S Sundaram ME Buchan SA Wilson SE Effectiveness of BNT162b2 and mRNA-1273 covid-19 vaccines against symptomatic SARS-CoV-2 infection and severe covid-19 outcomes in Ontario, Canada: test negative design study. BMJ. 2021;374:n1943. 10.1136/bmj.n1943 34417165 PMC8377789

[24] Stowe J, Andrews N, Gower C, Gallagher E, Utsi L, Simmons R, et al. Effectiveness of COVID-19 vaccines against hospital admission with the Delta (B.1.617.2) variant. Public library. London: UK Health Security Agency. [Accessed: 1 Jan 2023]. Preprint. Available from: https://khub.net/web/phe-national/public-library/-/document_library/v2WsRK3ZlEig/view_file/479607329?_com_liferay_document_library_web_portlet_DLPortlet_INSTANCE_v2WsRK3ZlEig_redirect=https%3A%2F%2Fkhub.net%3A443%2Fweb%2Fphe-national%2Fpublic-library%2F-%2Fdocument_library%2Fv2WsRK3ZlEig%2Fview%2F479607266

[25] Glatman-Freedman A Bromberg M Dichtiar R Hershkovitz Y Keinan-Boker L . The BNT162b2 vaccine effectiveness against new COVID-19 cases and complications of breakthrough cases: A nation-wide retrospective longitudinal multiple cohort analysis using individualised data. EBioMedicine. 2021;72:103574. 10.1016/j.ebiom.2021.103574 34537449 PMC8445746

[26] Gram MA Emborg HD Schelde AB Friis NU Nielsen KF Moustsen-Helms IR Vaccine effectiveness against SARS-CoV-2 infection or COVID-19 hospitalization with the Alpha, Delta, or Omicron SARS-CoV-2 variant: A nationwide Danish cohort study. PLoS Med. 2022;19(9):e1003992. 10.1371/journal.pmed.1003992 36048766 PMC9436060

[27] Skowronski DM Setayeshgar S Zou M Prystajecky N Tyson JR Galanis E Single-dose mRNA vaccine effectiveness against severe acute respiratory syndrome coronavirus 2 (SARS-CoV-2), including Alpha and Gamma variants: a test-negative design in adults 70 years and older in British Columbia, Canada. Clin Infect Dis. 2022;74:(7):1158-65. 34244723 10.1093/cid/ciab616 PMC8406884

[28] Skowronski DM Setayeshgar S Zou M Prystajecky N Tyson JR Sbihi H Comparative single-dose mRNA and ChAdOx1 vaccine effectiveness against severe acute respiratory syndrome coronavirus 2, including variants of concern: test-negative design, British Columbia, Canada. J Infect Dis. 2022;226(1):485-96. 10.1093/infdis/jiac023 35084500 PMC8807316

[29] Starrfelt J Danielsen AS Buanes EA Juvet LK Lyngstad TM Rø GØI Age and product dependent vaccine effectiveness against SARS-CoV-2 infection and hospitalisation among adults in Norway: a national cohort study, July-November 2021. BMC Med. 2022;20(1):278. 10.1186/s12916-022-02480-4 36050718 PMC9436448

[30] Vokó Z Kiss Z Surján G Surján O Barcza Z Wittmann I Effectiveness and waning of protection with different SARS-CoV-2 primary and booster vaccines during the Delta pandemic wave in 2021 in Hungary (HUN-VE 3 study). Front Immunol. 2022;13:919408. 10.3389/fimmu.2022.919408 35935993 PMC9353007

[31] Puranik A, Lenehan PJ, Silvert E, Niesen MJM, Corchado-Garcia J, O’Horo JC, et al. Comparison of two highly-effective mRNA vaccines for COVID-19 during periods of Alpha and Delta variant prevalence. medRxiv; 2021.08.06.21261707. 10.1101/2021.08.06

[32] Skowronski DM Febriani Y Ouakki M Setayeshgar S El Adam S Zou M Two-dose severe acute respiratory syndrome coronavirus 2 vaccine effectiveness with mixed schedules and extended dosing intervals: test-negative design studies from British Columbia and Quebec, Canada. Clin Infect Dis. 2022;75(11):1980-92. 10.1093/cid/ciac290 35438175 PMC9047203

[33] Goldberg Y Mandel M Bar-On YM Bodenheimer O Freedman L Haas EJ Waning immunity after the BNT162b2 vaccine in Israel. N Engl J Med. 2021;385(24):e85. 10.1056/NEJMoa2114228 34706170 PMC8609604

[34] Andrews N Tessier E Stowe J Gower C Kirsebom F Simmons R Duration of protection against mild and severe disease by Covid-19 vaccines. N Engl J Med. 2022;386(4):340-50. 10.1056/NEJMoa2115481 35021002 PMC8781262

[35] Feikin DR Higdon MM Abu-Raddad LJ Andrews N Araos R Goldberg Y Duration of effectiveness of vaccines against SARS-CoV-2 infection and COVID-19 disease: results of a systematic review and meta-regression. Lancet. 2022;399(10328):924-44. 10.1016/S0140-6736(22)00152-0 35202601 PMC8863502

