# Supplementary material for: Vaccine effectiveness against COVID-19 hospitalisation in adults (≥ 20 years) during Alpha- and Delta-dominant circulation: I-MOVE-COVID-19 and VEBIS SARI VE networks, Europe, 2021
Source: Euro Surveill. 2023 Nov 23;28(47):2300186. doi: 10.2807/1560-7917.ES.2023.28.47.2300186 (PMC10668259; doi:10.2807/1560-7917.ES.2023.28.47.2300186)
Supplement: Supplement [file 23-00186_ROSE_Supplement.pdf]

This supplementary material is hosted by *Eurosurveillance* as supporting information alongside the article “Vaccine effectiveness against COVID-19 hospitalisation in adults (≥ 20 years) during Alpha- and Delta-dominant circulation: I-MOVE-COVID-19 and VEBIS SARI VE networks, Europe, 2021”, on behalf of the authors, who remain responsible for the accuracy and appropriateness of the content. The same standards for ethics, copyright, attributions and permissions as for the article apply. Supplements are not edited by *Eurosurveillance* and the journal is not responsible for the maintenance of any links or email addresses provided therein.

Note about distribution of vaccinated patients across sites

The 12 completely vaccinated cases were distributed across five of the eight sites providing data during the Alpha period, while the partially vaccinated cases were found in all eight sites. Partially and completely vaccinated controls were from all eight sites, except for one which had no completely vaccinated SARI patients during the Alpha period.

Supplementary Table S1. Sensitivity analyses: Effectiveness of COVID-19 complete primary series vaccination (PSV) with and without mRNA booster dose against hospitalisation among adults (≥20 years) in patients with (a) no known prior infection and (b) severe outcomes only, all vaccine products combined, Alpha- and Delta-dominant periods, I-MOVE-COVID-19 and VEBIS hospital networks, Europe, 13 Dec 2021–31 Jul 2022

**Analysis (a1): Alpha period. Vaccine effectiveness of partial and complete PSV (all products combined), overall and by age group; SARI patients with no known prior infection**				
	**Vaccinated/unvaccinated cases; Vaccinated/unvaccinated controls**		**VE[a] (95% CI)**	
	**Partial PSV**	**Complete PSV**	**Partial PSV**	**Complete PSV**
**Any PSV product**	**7 sites;[b] N=1378[c]**	**7 sites;[b] N=1330[c]**		
All ≥20	56/891; 92/339	12/891; 88/339	45 (15–64)	86 (71–93)
Age group				
20–59	*6/200; 3/59*	1/200; 4/59	NC[d]	NC[d]
60–79	30/490; 54/177	1/490; 13/177	56 (19–76)	NC[d]
≥80	20/201; 35/103	10/201; 71/103	33 (-44–69)	77 (44–90)

**Analysis (a2): Delta period. Vaccine effectiveness of complete PSV regardless of time since last PSV dose, and PSV plus mRNA booster ≥150 days from last PSV dose (all products combined), overall and by age group; SARI patients with no known prior infection**				
	**Vaccinated/unvaccinated cases; Vaccinated/unvaccinated controls**		**VE[a] (95% CI)**	
	**Complete PSV only**	**Complete PSV plus mRNA booster**	**Complete PSV only**	**Complete PSV plus mRNA booster**
**Any PSV product**	**10 sites;[e] N=2481[f]**			
All ≥20	503/753; 1005/220	NC[g]	80 (74–84)	NC[g]
Age group				
20–59	102/420; 217/84	NC[g]	88 (82–92)	NC[g]
60–79	233/239; 465/87	NC[g]	79 (70–85)	NC[g]
≥80	168/94; 323/49	NC[g]	34 (-15–62)	NC[g]

**Analysis (b1): Alpha period. Vaccine effectiveness (VE) of partial and complete primary series vaccination (PSV; all products combined), overall and by age group; SARI patients with severe outcomes only (admitted to intensive care or in-hospital death)**

	Vaccinated/unvaccinated cases; Vaccinated/unvaccinated controls		VE[a] (95% CI)	
	**Partial PSV**	**Complete PSV**	**Partial PSV**	**Complete PSV**
**Any PSV product**	**5 sites;[i] N=341[j]**	**5 sites;[i] N=330[j]**		
All ≥20	14/290; 7/30	2/290; 8/30	NC[h]	NC[d]
Age group				
20–59	2/55; 0/5	0/55; 0/5	NC[d]	NC[d]
60–79	5/164; 4/14	0/164; 2/14	NC[d]	NC[d]
≥80	7/71; 3/11	2/71; 6/11	NC[d]	NC[d]

Analysis (b2): Delta period. Vaccine effectiveness (VE) of primary series vaccination (PSV) received ≥150 days prior to onset and PSV plus mRNA booster dose, with booster received ≥150 days from receipt of last PSV dose (all products combined), all ≥20 years; SARI patients with severe outcomes only (admitted to intensive care or in-hospital death)

	Vaccinated/unvaccinated cases; Vaccinated/unvaccinated controls		VE[a] (95% CI)	
	**Complete PSV only**	**Complete PSV plus mRNA booster**	**Complete PSV only**	**Complete PSV plus mRNA booster**
**Any PSV product**	**7 sites;[k] N=78[l]**	**3 sites;[g] N=42[l]**		
All ≥20	16/30; 26/6	3/28; 7/4	84 (20–97)	NC[d]

Analysis (b3): Delta period. Vaccine effectiveness (VE) of primary series vaccination (PSV) received ≥14 days prior to onset (all products combined), overall and by age group; SARI patients with severe outcomes only (admitted to intensive care or in-hospital death)

	Vaccinated/unvaccinated cases; Vaccinated/unvaccinated controls	VE[a] (95% CI)
	**Complete PSV only**	**Complete PSV only**
**Any PSV product**	**8 sites;[m] N=117[n]**	
All ≥20	35/34; 42/6	80 (37–94)

I-MOVE: Influenza – Monitoring vaccine effectiveness in Europe; PSV: primary series vaccination; VE: vaccine effectiveness; VEBIS: Vaccine Effectiveness, Burden and Impact Studies.

[a]Odds ratio adjusted by country, time (restricted cubic spline of swab date or swab month as categorical variable, depending on model), age (restricted cubic spline or age as linear variable, depending on model), sex, presence/absence of chronic condition (asthma, diabetes, heart disease, lung disease).

[b]Seven sites: Belgium, Croatia, France, Lithuania, Navarra, the Netherlands and Spain.

[c]N=1378 and 1330 after dropping all records of patients with known prior infection in those with partial primary series vaccination (PSV) only, and in those with complete PSV, respectively.

[d] Numbers are too small to provide robust VE estimates (<20 total vaccinated cases and controls).

[e]Ten sites: Belgium, Croatia, France, Ireland, Lithuania, Malta, Navarra, the Netherlands, Portugal and Spain.

[f]N=2481 after dropping all records of patients who did not known prior infection, in those with complete PSV only.

[g]Two of the three study sites with data in this category had no vaccinated cases, so this sensitivity analysis could not be performed.

[h]Numbers are too small to provide robust VE estimates (penalised logistic regression showed evidence of small sample bias).

[i]Five sites: Belgium, Croatia, Navarra, the Netherlands and Spain.

[j]N=341 and 330 after dropping all records of patients who did not have a severe outcome (or with this information missing), in those with partial and complete PSV, respectively.

[k]Seven sites: Belgium, France, Malta, Navarra, the Netherlands, Portugal and Spain.

[l]N=78 after dropping all records of patients who did not have a severe outcome (or with this information missing).

[m]Eight sites: Belgium, France, Ireland, Malta, Navarra, the Netherlands, Portugal and Spain.

[n]N=117 after dropping all records of patients who did not have a severe outcome (or with this information missing).

Supplementary Table S2. Effectiveness of COVID-19 partial and complete primary series vaccination (PSV) against hospitalisation among adults (≥20 years) in the PSV target group

during Alpha and Delta periods, by dose, age group and vaccine product, I-MOVE-COVID-19 and VEBIS hospital networks, Europe, March–December 2021 (N=4736)*

Analysis 1: Vaccine effectiveness of partial and complete PSV, by vaccine product and age group, Alpha period (March–June 2021; N=1625)

PSV vaccine product	Vaccinated/unvaccinated cases; vaccinated /unvaccinated controls		VE[a] (95% CI)			
	Partial PSV	Complete PSV	Partial PSV		Complete PSV	
**All products combined**	**7 sites;[b] N=1523[c]**	**7 sites;[b] N=1469[d]**				
All ≥20 years	62/1013; 94/354	12/1013; 90/354	43	(13 to 62)	86	(71 to 93)
Age group						
20–59	8/238; 3/62	1/238; 4/62	NC[e]	NC[e]	NC[e]	NC[e]
60–79	33/557; 55/183	1/557; 13/183	56	(21 to 76)	NC[e]	NC[e]
≥80	21/218; 36/109	10/218; 73/109	29	(-49 to 66)	77	(44 to 90)
Chronic condition[f]						
No	22/457; 16/101	2/457; 22/101	18	(-89 to 64)	95	(75 to 99)
Yes	40/556; 78/253	10/556; 68/253	47	(14 to 68)	80	(57 to 91)
**Comirnaty PSV**	**7 sites;[b] N=1463**	**6 sites;[g] N=1434**				
All ≥20 years	33/1013; 63/354	12/991; 82/349	45	(8 to 67)	85	(69 to 92)
Age group						
20–59	3/238; 3/62	1/225; 2/61	NC[e]		NC[e]	NC[e]
60–79	14/557; 30/183	1/548; 9/181	56	(4 to 80)	NC[e]	NC[e]
≥80	16/218; 30/109	10/218; 71/107	22	(-70 to 65)	75	(42 to 90)
Chronic condition[f]						
No	9/457; 10/101	2/445; 19/101	NC[e]		94	(68 to 99)
Yes	24/556; 53/253	10/546; 63/248	47	(4 to 70)	80	(56 to 91)
**Vaxzevria PSV**	**6 sites;[g] N=1358**	**0 sites;[h] N=0**				
All ≥20	21/975; 19/343	NC	31	(-46 to 67)		NC
Age group						
20–59	4/229; 0/60	NC	NC[e]	NC		NC
60–79	15/538; 18/180	NC	45	(-34 to 78)		NC
≥80	2/208; 1/103	NC	NC[e]	NC		NC
Chronic condition[f]						
No	10/442; 4/97	NC	NC[e]	NC		NC
Yes	11/533; 15/246	NC	48	(-33 to 80)		NC

Analysis 2: Vaccine effectiveness (VE) in patients eligible for a booster dose. Group A: those who received complete primary series vaccination (PSV) only, with last PSV dose administered ≥ 150 days before onset. Group B: those who received PSV plus an mRNA booster dose administered ≥ 150 days after last PSV dose (June–December 2021; N=3111)

PSV vaccine product	Vaccinated/unvaccinated cases; vaccinated /unvaccinated controls		VE[a] (95% CI)			
	Complete PSV (Group A)	Complete PSV + mRNA booster (Group B)	Complete PSV (Group A)		Complete PSV + mRNA booster (Group B)	
**All products combined**	**7 sites;[i] N=524[j]**	**2 sites;[k] N=88[l]**				
All ≥20 years	103/102; 268/51	4/39; 21/24	52	(18 to 71)	91	(57 to 98)
Age group						
20–59	10/45; 13/14	0/16; 1/9	62	(-77 to 92)	NC[e]	NC[e]
60–79	43/41; 105/24	1/13; 8/13	68	(30 to 85)	NC[e]	NC[e]
≥80	50/16; 150/13	3/10; 12/2	51	(-49 to 84)	NC[e]	NC[e]
Chronic condition[f]						

No	19/48; 51/13	1/19; 4/4	59	(-32 to 87)	NC[e]	NC[e]
Yes	84/54; 217/38	3/20; 17/20	50	(8 to 73)	95	(53 to 99)
**Comirnaty PSV**	**6 sites;[m] N=435**	**2 sites;[k] N=88**				
All ≥20 years	80/75; 233/47	4/39; 21/24	54	(18 to 74)	91	(57 to 98)
Age group						
20–59	4/34; 8/13	0/16; 1/9	NC[e]		NC[e]	NC[e]
60–79	29/34; 86/22	1/13; 8/13	75	(40 to 89)	NC[e]	NC[e]
≥80	47/7; 139/12	3/10; 12/2	40	(-97 to 82)	NC[e]	NC[e]
Chronic condition[f]						
No	12/29; 43/12	1/19; 4/4	53	(-86 to 88)	NC[e]	NC[e]
Yes	68/46; 190/35	3/20; 17/20	54	(10 to 76)	95	(53 to 99)

I-MOVE: Influenza – Monitoring vaccine effectiveness in Europe; PSV: primary series vaccination; VE: vaccine effectiveness; VEBIS: Vaccine Effectiveness, Burden and Impact Studies.

*Analyses 1 and 2 including stratified estimates.

[a]Odds ratio adjusted by study site as a fixed effect, time (restricted cubic spline of swab date, or swab month as a categorical term, depending on model), age or 5-year age group (linear or categorical term depending on model), sex, and presence of at least one of four chronic conditions (asthma, diabetes, heart disease, lung disease).

[b]Seven study sites: Belgium, Croatia, France, Lithuania, Navarra, the Netherlands and Spain.

[c]N=1523 after dropping 102 records from patients with complete vaccination.

[d]N=1469 after dropping 156 records from patients with partial vaccination.

[e]Numbers are too small to provide robust VE estimates when there are ≤ 20 vaccinated cases and controls in total.

[f]In this analysis stratified by chronic condition, the adjustment for presence of at least one chronic condition was removed.

[g]Six study sites: Belgium, Croatia, France, Navarra, the Netherlands and Spain.

[h]None of the participating sites with patients receiving complete vaccination had used Vaxzevria during the Alpha-dominant period.

[i]Seven study sites: Belgium, France, Malta, Navarra, the Netherlands, Portugal and Spain.

[j]N=524 after dropping 2195 records from patients who were not yet in the target group for booster dose vaccination, 220 from those vaccinated <150 days before onset, 107 from those with a booster dose, 106 from patients with partial vaccination, and 124 from sites with <5 cases/controls (or a total of cases and controls <20).

[k]Two study sites: France and Spain.

[l]N=88 after dropping 2195 records from patients who were not yet in the target group for booster dose vaccination, 634 from those with partial vaccination or complete vaccination without booster, 114 from sites with <5 cases/controls (or a total of cases and controls <20) and 80 from one site with no vaccinated cases.

[m]Six study sites: France, Malta, Navarra, the Netherlands, Portugal and Spain.

Supplementary Table S3. Target group for primary course vaccination

Target group for primary course vaccination	Country and date of eligibility								
	**Belgium**	**Spain**	**France**	**Croatia**	**Ireland**	**Lithuania**	**Malta**	**the Netherlands**	**Portugal**
Staff [a] and residents in a care home (RCH) for older adults	05/01/2021	27/12/2020	27/12/2020	27/12/2020		12/01/2021	27/12/2020		
Staff [a] and RCH for older adults and their carers (adults aged ≥65 years who are residents of long-term care facilities (LTCF))					04/01/2021				
Carers working in care homes for older and vulnerable adults[a]					04/01/2021			06/01/2021	
RCH for older adults and their carers;[a] professionals and users of the National Integrated Continued Care Network[a]									05/01/2021
Disabled people accommodated in specialised establishments and their staff[a] aged ≥50 years and/or with co-morbidities			27/12/2020						
Frontline health care workers (HCW; working in direct COVID care)								06/01/2021	
Frontline health and social care workers (HSCW) aged ≥50 years or presenting comorbidities			04/01/2021						
Frontline HSCW		01/01/2021		27/12/2020	29/12/2020	28/12/2020			27/12/2020
Frontline HSCW in hospitals	08/01/2021								
Frontline HSCW in collective institutions and hospital staff other than para/medical[a]	28/01/2021								
Frontline HSCW in primary care[a]	23/02/2021								
Other HSCW not considered frontline[a]		21/01/2021							

Frontline HSCW regardless of their age		06/02/2021						
HCW and LTCF workers[a] (public and private sector)						27/12/2020		
Mandatory vaccination for frontline HSCW		15/09/2021						
Adult working individuals (18–55 years) with an essential society role (police, army, public health and emergency response, teachers, school staff)[a]	09/02/2021							
All other frontliners (other health care professionals including pharmacists, university staff, water service and WasteServ employees, police officers, Armed Forces Malta, Civil Protection Department)[a]						01/02/2021		
Teachers, students[a]					08/02/2021			
Staff at schools and child-care centres[a]						22/02/2021		27/03/2021
Military[a]	09/02/2021				23/04/2021	01/02/2021		04/02/2021
Professionals in the armed forces, security forces and critical services[a]								04/02/2021
Clinically extremely vulnerable (CEV) individuals	21/01/2021	18/01/2021		10/03/2021	28/12/2020			
Individuals aged ≥16 years or parents of individuals under 16 years of age[a] with chronic illness whose state of health makes them particularly at risk of severe disease or death (insulin-dependent diabetics;[a] patients who are immunosuppressed; cancer patients undergoing chemotherapy;[a] people who have been treated for cancer in the last six months;[a] patients on dialysis;[a] those admitted to hospital for respiratory problems; patients suffering from cardiac disease or who attend the heart failure clinic[a]; people with Down's syndrome; [a] people who use a BiPap machine[a])						08/02/2021		
Other CEV individuals (various sub groups, starting with home-dwelling people with Down’s syndrome and morbid obesity[a])							15/02/2021	

People with Down's syndrome, aged ≥16 years[a]									24/03/2021
People aged ≥50 years with comorbidities			19/02/2021						
All individuals aged 16–64 years with underlying health conditions which put them at higher risk of serious disease and mortality	15/04/2021	01/04/2021		23/02/2021		08/03/2021			
People aged 16–59 years who have an underlying condition that puts them at high risk of severe disease and death					22/04/2021				
People aged 50–79 years of age with at least one of: coronary heart disease, renal insufficiency, COPD or chronic respiratory disease under ventilatory support and/or long-term oxygen therapy									05/01/2021
All those aged ≥18 years with comorbidities			01/05/2021						
People aged ≥16 years, with at least one of: diabetes, obesity (BMI > 35), active malignant neoplasm under treatment, candidates and people with transplanted organs, immunosuppression and primary immunodeficiencies, some severe neurologic diseases, [a] some severe mental disorders (namely psychosis), [a] chronic kidney disease, heart failure (including congenital heart disease) and coronary artery disease, chronic pulmonary disease (many causes), lysosomal storage diseases[a]									10/05/2021
Pregnant women between 14 and 36 weeks pregnant offered vaccine					28/05/2021				
Birth year 1930 and earlier								26/01/2021	
Birth year 1931–1935								29/01/2021	
All those aged ≥85 years							27/12/2020		
Birth year 1936-1940								05/02/2021	
All those aged ≥80 years		01/01/2021				15/02/2021	01/02/2021		03/02/2021
Birth year 1941–1945								06/03/2021	

All those aged ≥75 years		07/03/2021	18/01/2021			08/02/2021			
Birth year 1946–1950								06/04/2021	
All those aged ≥70 years		01/04/2021	27/03/2021		15/02/2021	03/03/2021	01/03/2021		
Birth year 1951–1955								19/04/2021	
All those aged ≥65 years	01/03/2021			23/02/2021		08/03/2021			30/03/2021
Birth year 1956–1960 invited by GP								15/02/2021	
All those aged ≥60 years					22/04/2021		01/03/2021		29/04/2021
Birth year 1961–1965								27/04/2021	
All those aged ≥55 years		22/03/2021	12/04/2021			10/05/2021			13/05/2021
Birth year 1966–1970								20/05/2021	
All those aged ≥50 years		07/04/2021	10/05/2021		15/05/2021		10/04/2021		24/05/2021
Birth year 1971–1975								26/05/2021	
All those aged ≥45 years					18/05/2021	17/05/2021			02/06/2021
Birth year 1976-1980								29/05/2021	
All those aged ≥40 years	08/05/2021	10/06/2021			02/06/2021		20/04/2021		15/06/2021
Birth year 1981–1985								01/06/2021	
All those aged ≥35 years					20/06/2021	24/05/2021			23/06/2021
Birth year 1986–1990								07/06/2021	
All those aged ≥33 years									28/06/2021
All those aged ≥30 years		01/07/2021			07/07/2021		04/05/2021		
Birth year 1991–1995								11/06/2021	
All those aged ≥25 years					16/07/2021				
All those aged ≥20 years		10/07/2021							

Birth year 1996–2003								15/06/2021	
All those aged ≥18 years	05/06/2021		12/05/2021		19/07/2021				04/07/2021
All those ≥18 years (for Pfizer ≥16 years)				01/05/2021					
All those aged ≥16 years					27/07/2021	31/05/2021	17/05/2021		
All those aged ≥15 years									13/07/2021
Birth year 2004–2009								22/06/2021	
All those aged ≥12 years	07/07/2021	25/07/2021	15/06/2021	27/09/2021	12/08/2021		16/06/2021		
All those aged ≥11 years									10/08/2021
Children and adolescents aged 12–17 with pre-existing conditions	05/06/2021								
All those aged 5–11 years of age at risk of severe COVID or in contact with immunocompromised people[a]			15/12/2021						
Birth year 2010–2016 with medical indication for vaccination								18/12/2021	
Birth year 2010–2016								18/01/2022	
All those aged ≥8 years									06/01/2022
All those aged ≥6 years									15/01/2022
All those aged ≥5 years	01/01/2022	15/12/2021		10/12/2021	10/01/2022		20/12/2021		22/01/2022
All those aged ≥12 years who are immunocompromised eligible for additional primary course dose					30/09/2021				
Third vaccination of people of aged ≥12 years with a serious immune disorder, who were under care of a medical specialist								06/10/2021	

Immunocompromised individuals: third dose as a part of primary vaccination; dose 3 to be given at least 4 weeks after dose 2 (preferably 8 weeks)	12/10/2021

[a]Insufficient information in dataset to code this target group

Supplementary Table S4. Target group for first booster vaccination

Target group for first booster vaccination	Date of eligibility in each country								
	**Belgium**	**Spain**	**France**	**Croatia**	**Ireland**	**Lithuania**	**Malta**	**the Netherlands**	**Portugal**
Mandatory delay from last primary course dose			5 months	3–6 months (2 months if primary vaccination Jcovden)		180 days			
Residents in residential structures for the elderly and similar institutions				12/10/2021					11/10/2021
Residents in a care home for older adults	06/10/2021	16/09/2021	01/09/2021	12/10/2021			06/09/2021	22/11/2021	
Institutionalised patients				12/10/2021		13/09/2021			
Other residents in long term care facilities		23/11/2021							
All those aged ≥65 years who are residents and staff[a] of long-term care facilities (LTCF)					27/09/2021				
All those 65 years of age and over and health professionals				12/10/2021					
Staff in a care home for older adults[a]	31/10/2021		01/09/2021	12/10/2021					
Health and social care workers (HSCW)	15/11/2021	23/11/2021		12/10/2021	25/11/2021	13/09/2021	08/11/2021	03/12/2021	
Frontline HSCW			06/10/2021						
Staff at schools and child-care centres[a]							15/11/2021		
Clinically extremely vulnerable individuals			01/09/2021	12/10/2021					
All those with increased medical risk[a]				12/10/2021				23/12/2021	
High-risk patients (immunosuppressive, after treatment,[a] haematologic cancer patients (on treatment or <24 months after treatment),						08/08/2021			

terminal CKD patients,[a] patients on dialysis[a]) final primary course dose vaccination at least 180 days prior									
Individuals with immune deficiency/ immunocompromised	09/09/2021			12/10/2021			13/09/2021		01/09/2021
Individuals in contact with immunocompromised patients[a]			06/10/2021	12/10/2021					
Adults with Down’s syndrome who live at home[a]								03/12/2021	
All those aged 16–59 years with underlying conditions and all residents of LTCF					15/11/2021				
People aged ≥12 years with a serious immune disorder, who were under care of a medical specialist				21/1/2022				06/01/2022	
All pregnant women aged ≥16 years					20/12/2021				
All those with primary series of vaccination with JCovden	15/11/2021	02/11/2021	01/09/2021	12/10/2021					
All those aged ≥18 years with primary series of vaccination with JCovden									23/11/2021
All with primary series of vaccination with Vaxzevria	15/11/2021	15/12/2021							
All those with final primary course dose at least 120 days prior for Comirnaty or JCovden, 180 days for Spikevax						17/11/2021			
Birth year 1930 and earlier								18/11/2021	
Birth year 1931–1935								19/11/2021	
Birth year 1936–1940								23/11/2021	
All those aged ≥80 years					27/09/2021				
Birth year 1941–1945								02/12/2021	

Birth year 1946–1950								08/12/2021	
All those aged ≥70 years		18/10/2021					27/09/2021		
Birth year 1951–1955								10/12/2021	
All those aged ≥65 years	04/10/2021	23/11/2021	01/09/2021			13/09/2021			11/10/2021
Birth year 1956–1960								12/12/2021	
All those aged ≥60 years		23/11/2021			05/11/2021		15/11/2021		
Birth year 1961–1965								14/12/2021	
All those aged ≥55 years									
Birth year 1966–1970								23/12/2021	
All those aged ≥50 years		15/12/2021			09/12/2021		29/11/2021		14/12/2021
Birth year 1971–1975								25/12/2021	
Birth year 1976–1980								26/12/2021	
All those aged ≥40 years		10/01/2022			19/12/2021				
Birth year 1981–1985								28/12/2021	
All those aged ≥35 years							20/12/2021		
Birth year 1986–1990								30/12/2021	
All those aged ≥30 years					29/12/2021				
Birth year 1991–1995								01/01/2022	
Birth year 1996–2003								02/01/2022	
All those aged ≥18 years	01/12/2021	13/01/2022	27/11/2021	07/09/2022			25/12/2021		22/12/2021
All those aged ≥16 years					02/01/2022				
Birth year 2004–2010								06/01/2022	
All those aged ≥12 years	01/03/2022				21/02/2022				

Children aged ≥12 years with increased risk for severe COVID-19 due to underlying medical conditions	07/09/2022	
Children aged 5–11 years who are immunocompromised		01/08/2022

[a]Insufficient information in dataset to code this target group.
